# Engagement and Utilization of a Complete Remote Digital Care Program for Musculoskeletal Pain Management in Urban and Rural Areas Across the United States: Longitudinal Cohort Study

**DOI:** 10.2196/44316

**Published:** 2023-03-16

**Authors:** Justin Scheer, Anabela C Areias, Maria Molinos, Dora Janela, Robert Moulder, Jorge Lains, Virgílio Bento, Vijay Yanamadala, Fernando Dias Correia, Fabíola Costa

**Affiliations:** 1 Department of Neurological Surgery University of California San Francisco, CA United States; 2 Sword Health Inc Draper, UT United States; 3 Institute for Cognitive Science University of Colorado Boulder Boulder, CO United States; 4 Rovisco Pais Medical and Rehabilitation Centre Coimbra Portugal; 5 Faculty of Medicine Coimbra University Coimbra Portugal; 6 Department of Neurosurgery Hartford Healthcare Medical Group Westport, CT United States; 7 Department of Surgery Frank H Netter School of Medicine Quinnipiac University Hamden, CT United States; 8 Neurology Department Centro Hospitalar e Universitário do Porto Porto Portugal

**Keywords:** physical therapy, physiotherapy, remote care, telerehabilitation, digital therapy, eHealth, telehealth, telemedicine, musculoskeletal, musculoskeletal conditions, urban, rural, pain, health inequity, digital care, pain management, clinical outcome, health equity, engagement

## Abstract

**Background:**

Musculoskeletal (MSK) conditions are the number one cause of disability worldwide. Digital care programs (DCPs) for MSK pain management have arisen as alternative care delivery models to circumvent challenges in accessibility of conventional therapy. Despite the potential of DCPs to reduce inequities in accessing care, the outcomes of such interventions in rural and urban populations have yet to be studied.

**Objective:**

The aim of this study was to assess the impact of urban or rural residency on engagement and clinical outcomes after a multimodal DCP for MSK pain.

**Methods:**

This study consists of an ad hoc analysis of a decentralized single-arm investigation into engagement and clinical-related outcomes after a multimodal DCP in patients with MSK conditions. Patients were coded according to their zip codes to a specific rural-urban commuting area code and grouped into rural and urban cohorts. Changes in their engagement and clinical outcomes from baseline to program end were assessed. Latent growth curve analysis was performed to estimate change trajectories adjusting for the following covariates: age, gender, BMI, employment status, and pain acuity. Outcomes included engagement, self-reported pain, and the results of the Generalized Anxiety Disorder 7-item, Patient Health Questionnaire 9-item, and Work Productivity and Activity Impairment scales. A minimum clinically important difference (MCID) of 30% was considered for pain.

**Results:**

Patients with urban and rural residency across the United States participated in the program (n=9992). A 73.8% (7378/9992) completion rate was observed. Both groups reported high satisfaction scores and similar engagement with exercise sessions, with rural residents showing higher engagement with educational content (*P*<.001) and higher program completion rates (*P*=.02). All groups showed a significant improvement in all clinical outcomes, including pain, mental health, and work productivity, without statistically significant intergroup differences. The percentage of patients meeting the MCID was similar in both groups (urban: 67.1%, rural: 68.3%; *P*=.30).

**Conclusions:**

This study advocates for the utility of a DCP in improving access to MSK care in urban and rural areas alike, showcasing its potential to promote health equity. High engagement, satisfaction, and completion rates were noted in both groups, as well as significant improvements in clinical outcomes.

**Trial Registration:**

ClinicalTrials.gov NCT04092946; https://clinicaltrials.gov/ct2/show/NCT04092946

## Introduction

Musculoskeletal (MSK) conditions are highly prevalent worldwide, resulting in significant disability and suffering [1], and were associated with up to US $380.9 billion of total medical expenditures in 2016 in the United States alone [2]. Exercise-based physical therapy is the mainstay treatment for such conditions [1,3-6]. Recently, telerehabilitation and digital physical therapy have emerged as alternative care delivery systems for a wide range of MSK conditions [7-10]. These alternative care delivery systems have shown to be effective and feasible compared to traditional physical therapy [11-18] while increasing access and affordability to patients and easing the burdens of conventional programs [19]. Accessibility is increased by reducing travel limitations and time barriers while eliminating any geographic restrictions. Additionally, digital therapy can increase compliance by allowing patients to undergo treatment at their convenience and at their own pace, increasing patient empowerment and self-management [8,10,20].

Despite the many benefits of telehealth, inequities in health other than underlying health status still exist based on age, geography, respective availability of health care facilities, and socioeconomic factors [21-27]. In fact, compared to those in urban areas, patients from rural areas tend to be older, are more likely to be obese, have higher rates of disability, have more chronic health conditions, and have higher fall rates [24,28]. Rural areas are known to have higher proportions of uninsured and underinsured individuals and higher costs of health care services when compared to urban areas [29]. Overall, 65% of rural US counties are designated as health professional shortage areas [30], and rural areas have lower patient-to-primary care physician ratios [31]. Patients in rural areas of the United States have fewer opportunities for in-person physical activity programs due to limited access to indoor facilities and limited transportation when compared to urban patients [32]. These inequities are further compounded by lower educational levels, higher rates of poverty, and lower rates of internet access in rural areas [24,33]. Also, individuals with limited or no digital literacy or with limited access to digital technology may not have the means to pursue and maintain a telehealth intervention [23]. It is therefore crucial to identify strategies for improved access and quality of physical therapy in these historically disinvested areas.

To our knowledge, no study has been conducted on the impact of urban or rural location on engagement and clinical outcomes following a telerehabilitation program for MSK conditions. We have previously reported a multimodal digital care program (DCP) combining exercise-based physical therapy with psychoeducational components, which provided a comprehensive approach to pain management. This program encourages patients to develop strategies and self-management skills to manage their pain and has been validated in several acute and chronic MSK conditions [15-17,34-36]. Additionally, the impact of race and ethnicity [37], as well as baseline mental health [38] and fear-avoidance beliefs [39], on final clinical outcomes have also been explored. The purpose of this study was to assess the impact of geographical location on engagement and clinical outcomes after a multimodal DCP, with the hypothesis being that patients from both rural and urban areas would have similar engagement and significant improvement in outcomes after program completion.

## Methods

### Study Design

This study is an ad hoc analysis of a decentralized, single-arm investigation into clinical and engagement-related outcomes following a multimodal DCP in patients with musculoskeletal (MSK) pain conditions. The DCP was administered at the patients’ homes and delivered between March 1, 2021, and March 10, 2022.

### Ethics Approval

This study is part of a trial that was prospectively registered on ClinicalTrials.gov (NCT04092946) on September 17, 2019, and approved by the New England Institutional Review Board (120190313) on June 18, 2020.

### Population

The study population included adults (≥18 years of age) who were beneficiaries of employer health plans from 50 US states and the District of Columbia. Employees and their dependents who reported either acute or chronic MSK pain in the spine, upper limbs, or lower limbs were eligible and were invited to apply to the DCP of Sword Health (located in Draper, Utah) through a dedicated website. Throughout enrollment, participants were asked to provide demographic data, including zip codes and baseline clinical information (eg, initial pain levels). Participants were informed about the study and invited to provide consent. The exclusion criteria were as follows: (1) a health condition (eg, cardiac or respiratory) not allowing a participant to engage in at least 20 minutes of light to moderate exercise, (2) being under treatment for active cancer, and (3) rapid loss of strength or numbness in the arms or legs or change in bowel or urinary function in the previous 2 weeks.

### Intervention

The DCP has been described previously [15-17,34-36]. In brief, this multimodal program consisted of 4-, 8-, or 12-week telerehabilitation interventions comprising exercise, education, and cognitive behavioral therapy (CBT). This program digitally interfaced between the patient and an assigned physical therapist (PT), who monitored the patient for the study duration. Participants who lacked internet access at home were given a Wi-Fi hotspot. A US Food and Drug Administration–listed class II medical device that consisted of inertial motion trackers, a mobile app in a dedicated tablet, and a cloud-based portal was made available to all patients. Briefly, the personalized exercises were displayed on the tablet, with trackers allowing real-time video and audio biofeedback on performance. At session end, the data related to the exercise sessions, such as compliance, presence or absence of movement errors, and level of pain and fatigue during the exercise, were registered and stored in a cloud-based portal. This portal enabled remote and asynchronous monitoring by the assigned PT, who revised the prescribed exercises if needed. Patients were recommended a frequency of 3 exercise sessions per week. The education and CBT components of the program were developed by a multidisciplinary team following current clinical guidelines and state-of-the-art research [40-44]. The education component delved into topics focused on anatomy, physiology, symptoms, evidence-based treatments, fear avoidance, and active coping skills (including managing feelings of anxiety and depression). The CBT program was based on third-generation techniques—mindfulness, acceptance, and commitment therapy; empathy-focused therapy; fear-avoidance behavior; and constructive coping. The education and CBT materials were delivered to the patients through written articles, audio content, and interactive modules. Bidirectional communication with the assigned PT was ensured through built-in secure chat within a smartphone app and through video calls. Participants who did not perform any exercise session for 28 consecutive days were considered dropouts.

### Demographic Data

Demographic data included age, BMI, patient gender, educational level, and employment status. The gender category included “man,” “woman,” “nonbinary,” “other,” and “prefer not to specify.” The employment status categories were defined as the following: full-time employed, part-time employed, or not employed. The educational levels were (1) high school or less (including technical or vocational training), (2) some college, including a bachelor’s degree, community college, or an associate degree, (3) some graduate school, including a master’s or doctoral degree, and (4) “not available” or “prefer not to answer.”

Patients were coded according to their zip codes to a specific rural-urban commuting area (RUCA) code [45]. RUCA codes characterize all census areas regarding their rural and urban status and relationships. This classification system uses the standard Bureau of Census urbanized area and urban cluster definitions in combination with work-commuting information [46,47]. Rural areas have been defined as having an urban core of 50,000 people or less [24]. Therefore, using primary RUCA codes, we defined urban areas by scores from 1 to 3, and rural areas by aggregating codes 4 to 10 (Multimedia Appendix 1, Table S1 [47]).

### Outcomes

Outcomes were collected at baseline and 4, 8, and 12 weeks, and mean changes were calculated between baseline and program end. Engagement and clinical outcomes are described in Table 1.

**Table 1:** Engagement and clinical outcomes in this study.

Outcome	Description
Engagement	Measured through the following: Completion of the program (considered as the retention rate) Number of completed exercise sessions over the 12-week digital care program Weekly session frequency Time spent performing exercise sessions Articles read Interactions with the physical therapist Satisfaction, assessed through the question “On a scale from 0 to 10, how likely is it that you would recommend this intervention to a friend or neighbor?”
Numerical Pain Rating Scale [48,49]	Assessed through the question “Please rate your average pain over the last 7 days, from 0 (no pain at all) to 10 (worst pain imaginable)”; the number of patients reaching the minimum clinically important difference of 30% between baseline and treatment end was also assessed
Generalized Anxiety Disorder 7-item scale (range 0-21) [50]	Used to assess anxiety; higher scores are associated with worse outcomes
Patient Health Questionnaire 9-item scale (range 0-27) [51]	Used to assess depression; higher scores are associated with worse outcomes
WPAI^a^ for general health questionnaire (version 2.0) [52]	Evaluated in employed participants to assess overall work impairment (WPAI overall: total presenteeism and absenteeism from work), presenteeism (WPAI work), absenteeism (WPAI time), and non–work-related activity impairment (WPAI activity); higher scores represent higher impairment.

^a^WPAI: Work Productivity and Activity Impairment.

### Statistical Analysis

Analyses of baseline characteristics (demographics and clinical data), as well as engagement metrics, were performed using a 2-tailed, 2-sample *t* test, a 2-way analysis of variance (ANOVA) with Bonferroni post hoc test, a chi-square test, or a 2-proportion *z* test. Patients who completed the program were defined as “completers” and those that did not were defined as “noncompleters.”

Latent growth curve analysis (LGCA) was used to estimate trajectories of each outcome over time [35,53]. The LGCA has been recognized as one of the most powerful methods to analyze longitudinal data, since it provides a measure of fitness and addresses missing data through full information maximum likelihood (FIML) [54-57]. FIML uses all available data at each time point from all participants to calculate maximum likelihood estimates, outperforming multiple imputation by chained equations or listwise deletion [58,59]. In addition, the LGCA uses a structural equation model to define trajectories through intercept, slope, and curvature for each variable, allowing analysis of the recovery pace and leveling of the effect for each outcome. In order to account for unbalanced group sizes, a multiple-group LGCA was conducted. This allows for creating separate models for rural and urban groups while simultaneously performing intergroup comparisons (eg, mean change). A conditional analysis was conducted to assess the influence of age, gender, BMI, employment status, and education level and was fitted as a random effect. Additionally, analysis of subpopulations was performed by focusing on participants who met the following criteria at baseline: Generalized Anxiety Disorder 7-item (GAD-7) and Patient Health Questionnaire 9-item (PHQ-9) scores equal or greater than 5 points [50,51] and a Work Productivity and Activity Impairment (WPAI; comprising overall, work, time, and activity) score greater than 0 points. A robust sandwich estimator was used in all models for standard errors.

All statistical analyses were conducted using commercially available software (SPSS version 22; IBM Corp), and the level of significance was set at *P*<.05 for all tests. The LGCA was coded using R (version 4.2.2; R Foundation for Statistical Computing).

## Results

### Participant Inclusion

**Figure 1:**
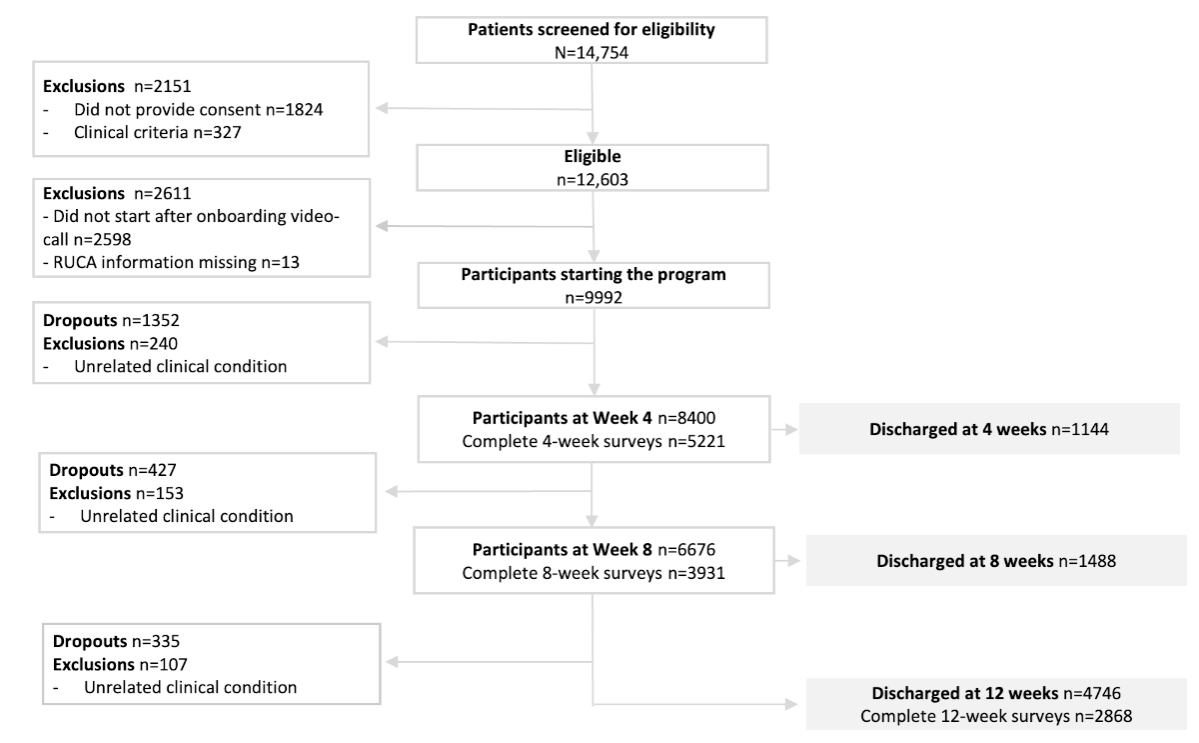
Study flow chart showing the number of participants who were excluded, included, and dropped out. RUCA: rural-urban commuting area.

A total of 14,754 participants were screened for eligibility (Figure 1). Of these, 2151 were excluded, for a total of 12,603 (85.4%) eligible patients, of whom 2611 were excluded due to unavailable RUCA data or not starting the program, resulting in a total of 9992 patients at program start. A total of 7378 of 9992 (73.8%) patients completed the program. The study flow diagram is presented in Figure 1.

### Baseline Characteristics

Patients’ baseline demographics grouped by urban and rural areas are presented in Table 2, while baseline characteristics stratified by completers and noncompleters can be found in Multimedia Appendix 1, Table S2.

**Table 2:** Baseline characteristics for urban and rural groups following an intention-to-treat analysis. Filtered cases correspond to participants who reported relevant impairment at baseline (>0 or ≥5 points). Statistically significant *P* values are italicized.

		Total (n=9992)	Urban (n=8809)	Rural (n=1183)	*P* value	
Age (years), mean (SD)		48.55 (12.45)	48.11 (12.37)	51.85 (12.57)	*<.001*	
**Age categories (years), n (%)**						*<.001*
	<25	127 (1.3)	114 (1.3)	13 (1.1)		
	25-40	2753 (27.6)	2520 (28.6)	233 (19.7)		
	40-60	5279 (52.8)	4649 (52.8)	630 (53.3)		
	>60	1833 (18.3)	1526 (17.3)	307 (26)		
BMI (kg/m^2^), mean (SD)		29.18 (6.74)	28.96 (6.60)	30.83 (7.48)	*<.001*	
**BMI categories (kg/m^2^), n (%)**						*<.001*
	Underweight (<18.5)	90 (0.9)	84 (1)	6 (0.5)		
	Normal (18.5-25)	2798 (28)	2548 (28.9)	250 (21.1)		
	Overweight (25-30)	3373 (33.8)	3011 (34.2)	362 (30.6)		
	Obese (30-40)	2957 (29.6)	2525 (28.7)	432 (36.5)		
	Obese grade III (>40)	743 (7.4)	614 (7)	129 (10.9)		
**Gender, n (%)**						.12
	Woman	5502 (55.1)	4818 (54.7)	684 (57.8)		
	Man	4457 (44.6)	3963 (45)	494 (41.8)		
	Nonbinary	24 (0.2)	19 (0.2)	5 (0.4)		
	Other	3 (0)	3 (0)	0 (0)		
	Prefer not to specify	6 (0.1)	6 (0.1)	0 (0)		
**Employment status, n (%)**						*<.001*
	Employed full-time	6271 (62.8)	5616 (63.8)	655 (55.4)		
	Employed part-time	2348 (23.5)	2076 (23.6)	272 (23)		
	Not employed	1067 (10.7)	853 (9.7)	214 (18.1)		
**Education level, n (%)**						*.001*
	High school or less	866 (8.7)	700 (7.9)	166 (14)		
	Some college, including bachelor’s or associate degree	4543 (45.5)	4031 (45.8)	512 (43.3)		
	Some graduate school, including master’s or doctoral degree	2082 (20.8)	1876 (21.3)	206 (17.4)		
	Not available or prefer not to answer	2501 (25)	2202 (25)	299 (25.3)		
**Acuity, n (%)^a^**						*<.001*
	Acute	2147 (21.5)	1952 (22.2)	195 (16.5)		
	Chronic	7845 (78.5)	6857 (77.8)	988 (83.5)		
**Anatomical pain region, n (%)**						*.003*
	Ankle	422 (4.2)	380 (4.3)	42 (3.6)		
	Elbow	286 (2.9)	259 (2.9)	27 (2.3)		
	Hip	900 (9)	786 (8.9)	114 (9.6)		
	Knee	1438 (14.4)	1292 (14.7)	146 (12.3)		
	Low back	3976 (39.8)	3441 (39.1)	535 (45.2)		
	Neck	936 (9.4)	834 (9.5)	102 (8.6)		
	Shoulder	1632 (16.3)	1461 (16.6)	171 (14.5)		
	Wrist or hand	402 (4)	356 (4)	46 (3.9)		
**Clinical outcomes (score)**						
	Pain, mean (SD)	4.83 (1.99)	4.83 (1.99)	4.85 (1.98)	.72	
	GAD-7^b^ ≥5, n (%)	2751 (27.5)	2430 (27.6)	321 (27.1)	.74	
	GAD-7 ≥5, mean (SD)	8.89 (4.08)	8.89 (4.07)	8.89 (4.19)	.99	
	GAD-7, mean (SD)	3.03 (4.35)	3.04 (4.35)	2.98 (4.37)	.69	
	PHQ-9^c^ ≥5, n (%)	2071 (20.7)	1790 (20.3)	281 (23.8)	*.006*	
	PHQ-9 ≥5, mean (SD)	9.21 (4.30)	9.20 (4.29)	9.31 (4.41)	.69	
	PHQ-9, mean (SD)	2.37 (4.15)	2.33 (4.11)	2.7 (4.41)	*.004*	
	WPAI^d^ overall >0, mean (SD)	29.89 (20.11)	29.9 (20.11)	29.80 (20.16)	.91	
	WPAI overall, mean (SD)	17.32 (21.26)	17.23 (21.25)	17.9 (21.39)	.34	
	WPAI work >0, mean (SD)	28.63 (18.80)	28.62 (18.77)	28.71 (19.05)	.91	
	WPAI work, mean (SD)	16.27 (20.05)	16.16 (20.0)	17.04 (20.35)	.21	
	WPAI time >0, mean (SD)	18.08 (18.07)	18.45 (18.45)	15.38 (14.94)	.11	
	WPAI time, mean (SD)	1.91 (8.07)	1.94 (8.22)	1.65 (6.82)	.31	
	WPAI activity >0, mean (SD)	37.37 (22.86)	37.33 (22.85)	37.70 (22.91)	.65	
	WPAI activity, mean (SD)	29.04 (25.46)	28.92 (25.45)	29.93 (25.48)	.20	
Medications, n (%)		2364 (23.7)	2062 (23.5)	302 (25.6)	.11	

^a^A total of 1.1% (114/9992) of patients were postsurgical.

^b^GAD-7: Generalized Anxiety Disorder 7-item scale.

^c^PHQ-9: Patient Health Questionnaire 9-item scale.

^d^WPAI: Work Productivity and Activity Impairment scale.

Patients from rural areas were significantly older than patients from urban areas (51.85, SD 12.57 years vs 48.11, SD 12.37 years, respectively; *P*<.001), had higher BMI (30.83, SD 7.48 kg/m^2^ vs 28.96, SD 6.60 kg/m^2^, respectively), had a higher percentage of unemployed individuals (214/1183, 18.1% vs 853/8809, 9.7%, respectively; *P*<.001), and had a higher percentage of individuals with a lower educational level (166/1183, 14% vs 700/8809, 7.9%, respectively; *P*<.001). No statistically significant differences in gender distribution were found between groups.

Patients in rural areas also presented a higher prevalence of chronic pain and low back pain conditions than patients from urban areas (535/1183, 45.2% vs 3441/8809, 39.1%, respectively). In opposition, a higher prevalence of knee-related conditions was observed in patients from urban areas than from rural areas (1292/8809, 14.7% vs 146/1183, 12.3%, respectively; *P*<.001).

Overall, similar clinical metrics were observed between patients from rural and urban areas at baseline. The statistically significant differences found were in baseline depression (rural PHQ-9 score 2.70, SD 4.41 vs urban PHQ-9 score 2.33, SD 4.22; *P*=.004) and percentage of patients with at least mild or moderate depression at baseline (281/1183, 23.8% vs 1790/8809, 20.3%, respectively; *P*=.006), where more patients from rural areas were depressed.

When comparing completers with noncompleters, the latter were younger (46.23, SD 12.61 years vs 49.38, SD 12.29 years, respectively; *P*<.001) and reported higher BMI (30.22, SD 7.36 kg/m^2^ vs 28.81, SD 6.47 kg/m^2^, respectively; *P*<.001). A larger proportion of noncompleters were employed full-time (1732/2614, 66.3% vs 4539/7378, 61.5%, respectively; *P*<.001) and reported a lower educational level. Noncompleters also reported higher levels of impairment in productivity (WPAI overall, *P*=.004 and WPAI work, *P*=.001) and non–work-related activities (WPAI activity, *P*=.01) at baseline.

### Engagement

Individuals from rural areas were more likely to complete the program than patients from urban areas (906/1183, 76.6% vs 6472/8809, 73.5%, respectively; *P*=.02). However, independently of dropout rates, both groups had a similar pattern of engagement. Engagement data stratified by patients from urban and rural areas is presented in Table 3. The 2 groups had similar time dedicated to exercise (*P*=.48), number of sessions (*P*=.77), sessions per week (*P*=.11), and interactions with the PT (*P*=.14). Average satisfaction scores were similarly high in both groups (rural score 8.6, SD 1.7 and urban score 8.6, SD 1.8; *P*=.95). The single significant difference in engagement between groups was the number of educational articles consulted, with patients from rural areas reading more articles than patients from urban areas (*P*<.001; Table 3).

**Table 3:** Engagement data across the groups. Statistically significant *P* values are italicized.

Engagement outcomes	Urban, mean (SD)	Rural, mean (SD)	*P* value
Sessions, n	33.88 (32.23)	34.18 (30.94)	.77
Sessions per week, n	2.76 (1.14)	2.78 (1.12)	.11
Training time, minutes	472.02 (485.56)	482.54 (485.96)	.48
Articles read, n	2.72 (5.27)	3.44 (6.18)	*<.001*
Interactions with physical therapist, n	11.79 (12.54)	12.37 (13.90)	.14
Average satisfaction score	8.6 (1.7)	8.6 (1.8)	.95

### Clinical Outcomes

**Figure 2:**
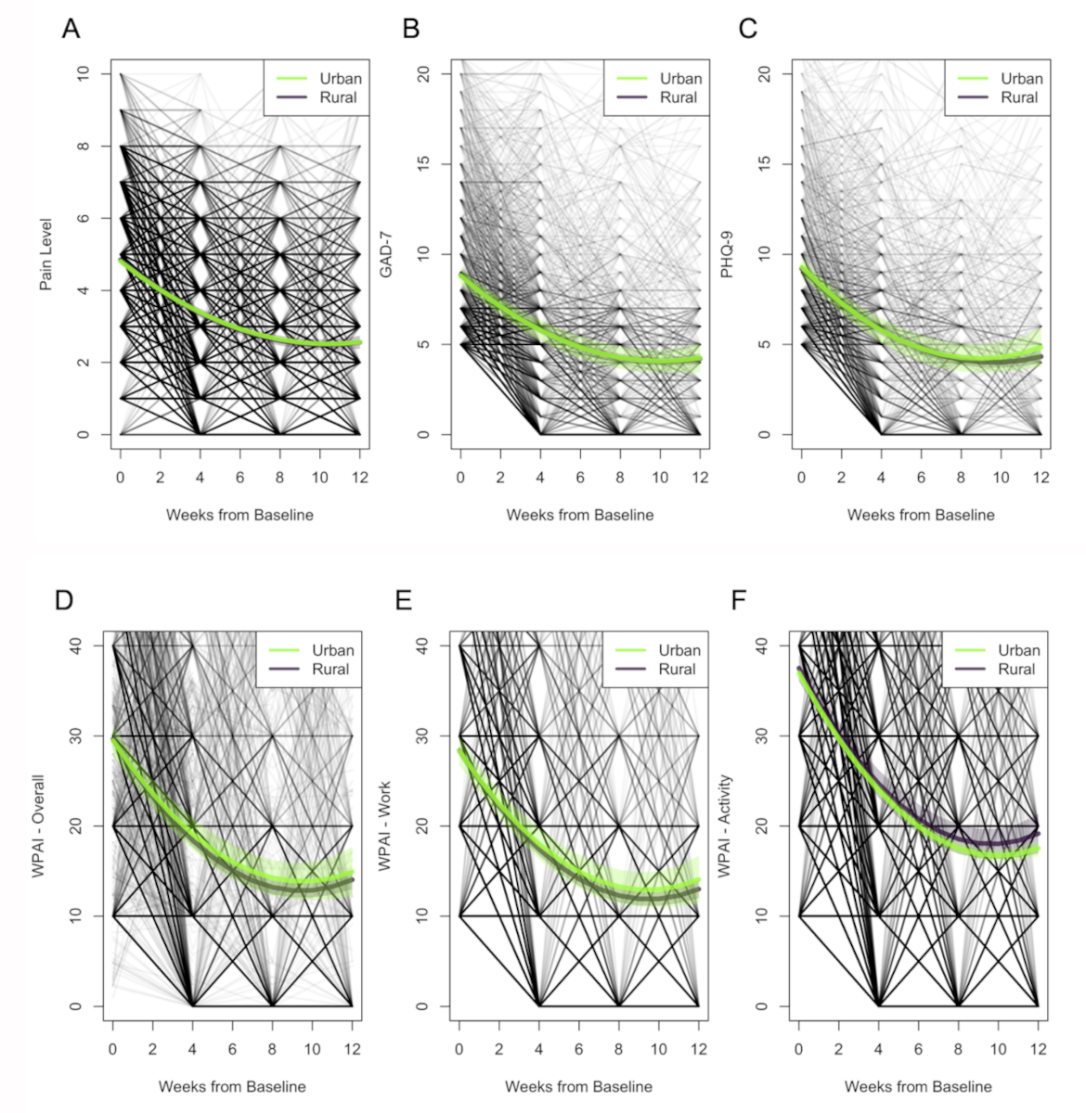
Longitudinal changes across time using intent-to-treat analysis. A: pain level; B and C: mental health (GAD-7-and PHQ-9 scores, respectively) for cases with at least mild or moderate anxiety or depression at baseline; D-F: work productivity (WPAI overall, WPAI work, and WPAI activity scores, respectively) for cases reporting impairment at baseline. The shaded areas are the 95% CI. GAD-7: Generalized Anxiety Disorder 7-item scale; PHQ-9: Patient Health Questionnaire 9-item scale; WPAI: Work Productivity and Activity Impairment scale.

Mean changes in clinical outcomes for both urban and rural groups following an intent-to-treat analysis are presented in Table 4, while the corresponding model estimates and model fitness are presented in Multimedia Appendix 1, Tables S3 and S4, respectively [60,61]. Change trajectories of each outcome are depicted in Figure 2. The impact of the covariates in clinical outcomes is presented in Multimedia Appendix 1, Table S5. The same analysis following a per-protocol approach is presented in Multimedia Appendix 1, Tables S6-S8. Similar results were observed from both intention-to-treat and per-protocol approaches. Since intention-to-treat analysis offers an overview change of the entire cohort, the following section is focused on these results.

**Table 4:** Mean changes between baseline and program end and mean differences between groups for the studied clinical outcomes following an intent-to-treat analysis. Statistically significant *P* values are italicized.

Scores	Urban		Rural			Mean difference	
	Mean change (95% CI)	*P* value	Mean change (95% CI)	*P* value	Difference (95% CI)		*P* value
Pain	2.2 (2.2 to 2.3)	*<.001*	2.3 (2.1 to 2.5)	*<.001*	–0.1 (–0.3 to 0.2)		.62
GAD-7^a^	1.26 (1.16 to 1.37)	*<.001*	1.16 (0.86 to 1.47)	*<.001*	0.1 (–0.22 to 0.43)		.53
GAD-7 ≥5	4.5 (3.7 to 5.4)	*<.001*	4.6 (4.3 to 4.9)	*<.001*	–0.1 (–1.0 to 0.8)		.85
PHQ-9^b^	0.93 (0.82 to 1.03)	*<.001*	1.14 (0.84 to 1.45)	*<.001*	–0.22 (–0.54 to 0.1)		.19
PHQ-9 ≥5	4.5 (3.39 to 5.53)	*<.001*	4.9 (4.5 to 5.2)	*<.001*	–0.41 (–1.5 to 0.7)		.48
WPAI^c^ overall	7.37 (6.65 to 8.09)	*<.001*	7.19 (5.28 to 9.11)	*<.001*	0.18 (–1.87 to 2.22)		.87
WPAI overall >0	14.6 (11.7 to 17.4)	*<.001*	15.6 (14.5 to 16.7)	*<.001*	–1.0 (–4.1 to 2.0)		.50
WPAI work	13.73 (12.99 to 14.47)	*<.001*	13.59 (11.67 to 15.51)	*<.001*	0.15 (–1.91 to 2.2)		.89
WPAI work >0	14.3 (11.6 to 17.0)	*<.001*	15.4 (14.3 to 16.4)	*<.001*	–1.1 (–4 to 1.8)		.46
WPAI time missed	7.14 (6.47 to 7.81)	*<.001*	6.82 (5 to 8.63)	*<.001*	0.32 (–1.62 to 2.25)		.75
WPAI time missed >0	11.4 (6.9 to 16.0)	*<.001*	11.8 (9.9 to 13.7)	*<.001*	–0.35 (–5.2 to 4.5)		.89
WPAI activity	0.66 (0.37 to 0.96)	*<.001*	0.42 (–0.34 to 1.19)	.28	0.24 (–0.58 to 1.06)		.57
WPAI activity >0	19.4 (18.6 to 20.3)	*<.001*	18.4 (16.1 to 20.7)	*<.001*	1.06 (–1.4 to 3.5)		.40

^a^GAD-7: Generalized Anxiety Disorder 7-item scale.

^b^PHQ-9: Patient Health Questionnaire 9-item scale.

^c^WPAI: Work Productivity and Activity Impairment scale.

#### Pain

Pain levels decreased similarly in both groups from baseline to program end (*P*=.62), corresponding to a significant mean change of 2.2 points (95% CI 2.2-2.3) in the urban cohort and 2.3 points (95% CI 2.1-2.5) in the rural cohort (Table 4 and Figure 2A). The rate of patients meeting pain MCID at program end was not statistically different between urban and rural cohorts (67.1% versus 68.3%, *P*=.30).

#### Mental Health

Among those who reported at least mild or moderate anxiety (GAD-7 score ≥5) at baseline, we observed a significant decrease in anxiety in both groups (urban score 4.5, 95% CI 3.7-5.4; *P*<.001 and rural score 4.6, 95% CI 4.3-4.9; *P*<.001); this decrease was similar in the groups (*P*=.85). Likewise, among those reporting at least mild or moderate depression at baseline (ie, PHQ-9 score ≥5), we observed a significant decrease in depression scores in both groups (urban score 4.5, 95% CI 3.39-5.53; *P*<.001 and rural score 4.9, 95% CI 4.5-5.2; *P*<.001); this decrease was also similar in the groups (*P*=.48). In patients from urban areas, chronic MSK conditions were associated with steeper initial recovery of depression, followed by a stronger leveling effect. In contrast, in patients from rural areas, chronic pain was associated with slower improvement that was more sustained over time (Figure 2C, difference in slope between groups –0.07, *P*=.004; difference in curve between groups 1.06, *P*<.001; Multimedia Appendix 1, Table S5).

#### Productivity

Productivity improvements were observed in both groups with no differences between them (Figure 2D-F). For WPAI overall, mean changes of 14.6 points (95% CI 11.7-17.4) and 15.6 points (95% CI 14.5-16.7) were reported for urban and rural groups, respectively (*P*=.50). Presenteeism, measured through WPAI work, improved by 14.3 points (95% CI 11.6-17.0) in the urban group and by 15.4 points (95% CI 14.3-16.4) in the rural group (*P*=.46). Absenteeism, measured through WPAI time missed, was reduced by 11.4 points (95% CI 6.9-16.0) and 11.8 points (95% CI 9.9-13.7) in the urban and rural groups, respectively. Similarly, impairment in non–work-related activities (ie, WPAI activity) had improvements of 19.4 points (95% CI 18.6-20.3) and 18.4 points (95% CI 16.1-20.7) in the urban and rural groups, respectively.

In urban areas, individuals with higher BMI reported a greater leveling effect on absenteeism improvement than those from rural areas (the difference in curve between groups was 0.14, *P*=.04; Multimedia Appendix 1, Table S5). In patients from rural areas, the presence of chronic pain was associated with a faster recovery in absenteeism in comparison with that in patients from urban areas (the difference in slope between groups was 0.16, *P*=.03; Multimedia Appendix 1, Table S5).

## Discussion

### Principal Findings

The multimodal DCP herein reported was able to reach all US states in both urban and rural locations and had a completion rate of 73.8% (7378/9992), which is similar to previous studies reporting the use of digital interventions for MSK pain management [37,62]. The percentage of participants in urban areas was significantly higher than in rural locations, which was expected given that only approximately 19.6% of the US population is located in rural areas according to the US Census Bureau [63].

Health inequities between urban and rural populations are prevalent in the United States. [64]. Rural populations have been reported to have demographics associated with a poorer prognosis for MSK pain [65-69]. In accord with this, in this study, patients from rural areas were older [67], had higher BMI [69], had lower educational levels [65], and had a higher prevalence of depression [67]. Some of these factors have previously been associated with lower chances of receiving physical therapy [33]. Lower education (and consequently lower digital literacy) have been associated with lower adherence, for example [21-23].

Studies have shown that patients in rural areas of the United States may face additional difficulties in recovery due to fewer opportunities for in-person physical activity programs as a consequence of limited access to indoor facilities, limited transportation, and a lower overall health status when compared to urban patients [24,32]. Additionally, rural residents are less likely to report having home broadband than those living in urban or suburban areas [70], which seriously impacts their access to digital health care tools and electronic communication with health providers [71].

In this study, engagement was similar between both rural and urban areas (eg, the number of sessions and interactions with a PT), and completion rates were higher in the rural cohort. The reasons behind these observations may be multifactorial, but one can speculate that the lack of access to alternative health care resources, as well as the provision of a Wi-Fi hotspot to those without internet, might have prompted patients from rural areas to not only engage with the exercise sessions but also to achieve higher completion rates [70,71]. Also, despite lower educational levels, patients from rural areas engaged more with curated health educational articles advocating for telerehabilitation programs as enablers of health literacy.

Despite the worse clinical outcomes reported at baseline by those in rural communities, in line with what has been described before [32,66-69], similar improvements in pain, mental health, and productivity impairment were observed in both groups, again reinforcing the notion that higher MSK pain burden in rural areas may be associated with lack of access to care. Pain improvements were above a 2-point change independently of the studied group, with 67.1% to 68.3% of participants meeting the MCID for pain [48,49]. The percentages of patients meeting the MCID were within the ranges previously reported for digital interventions (49%-75.6%) [72-74] and in-person physical therapy [75].

The prevalence of depression and anxiety has been reported to be higher in residents of rural areas compared to urban areas [76], with those from rural areas facing a shortage of mental health services [77]. Since mental health and MSK pain are tightly associated [38,78], the scarcity of psychological support can seriously impact the recovery rates of rural populations. This study confirms a higher depression burden in patients from rural areas but found similar improvement in mental health scores in both rural and urban patients, reinforcing the notion that lack of access to mental health resources may be the main driver for the higher burden of disease in rural areas. Additionally, MSK pain has been reported to be a main driver for loss of work productivity [79,80]. The factors weighing on absenteeism recovery were BMI and the presence of chronic pain, both previously reported to negatively impact MSK pain recovery [81,82]. Nevertheless, we did not observe significant differences in improvement in any productivity domains between urban and rural groups; all of these domains showed significant improvement following the DCP.

Despite the wide reach of telerehabilitation, many areas across the United States are still facing unmet needs. The results observed herein support the need for further research and investment in digital rehabilitation to mitigate inequities in health care access and care delivery optimization.

### Strengths and Limitations

There are many strengths to this study, namely the novelty of investigating the urban-rural dichotomy within a digital therapy program in a large sample size from a real-world context, including patients from 50 US states and the District of Columbia, which allows for a diverse population and thus better generalizability. Another strength is the DCP itself, which uses a multimodal approach that includes exercises with real-time biofeedback, mental support, regular communication with the PT, and a digital format. All these components favor accessibility and maximize engagement and clinical outcomes, allowing us to study different aspects of the problem, from pain to mental health to productivity.

The classification of rural and urban areas is a challenging topic considering the multitude of factors that can highly influence the obtained readings. Despite the application of a recognized classification system [45-47], we cannot rule out the existence of other confounding factors with contributions that were not taken into account during this exploratory analysis, including desirability bias. Other limitations include the lack of control groups (to account for nonspecific treatment effects) and the lack of long-term outcome and objective outcome measures (ie, through activity trackers). Nevertheless, this exploratory study may lay a foundation for future work in this field, identifying areas in need of improvement for future telerehabilitation programs. Further prospective controlled studies are warranted to better characterize the effect of rural and urban inequities on digital therapy outcomes.

### Conclusion

This study provides important insights regarding the impact of a multimodal digital program for MSK pain management in rural and urban settings. The DCP was able to reach all areas across the United States with high completion rates in both settings. Despite the inherent health inequities between patients from rural and urban areas, similarly high satisfaction and engagement, alongside significant improvements in pain, mental health, and productivity, were observed in both groups. This showcases the potential of the DCP to mitigate inequities by improving the accessibility of MSK care independently of geographic location.

## Appendix

Multimedia Appendix 1 Information regarding (1) Rural-urban commuting area (RUCA) codes, (2) baseline characteristics of completers and non-completers, (3) Latent growth curve analysis (LGCA) model following intent-to-treat analysis with corresponding model fitness and conditional analysis, and (4) 12-week mean changes for the per-protocol analysis and corresponding LGCA model and model fitness.

## Abbreviations

ANOVA: analysis of variance
CBT: cognitive behavioral therapy
DCP: digital care program
FIML: full information maximum likelihood
GAD-7: Generalized Anxiety Disorder 7-item
LGCA: latent growth curve analysis
MCID: minimum clinically important difference
MSK: musculoskeletal
PHQ-9: Patient Health Questionnaire 9-item
PT: physical therapist
RUCA: rural-urban commuting area
WPAI: Work Productivity and Activity Impairment

## Data Availability

The data sets generated during or analyzed during this study are available from the corresponding author upon reasonable request.
