# Supplementary material for: Engagement and Utilization of a Complete Remote Digital Care Program for Musculoskeletal Pain Management in Urban and Rural Areas Across the United States: Longitudinal Cohort Study
Source: JMIR Mhealth Uhealth. 2023 Mar 16;11:e44316. doi: 10.2196/44316 (PMC10132051; doi:10.2196/44316)
Supplement: Multimedia Appendix 1 [file mhealth_v11i1e44316_app1.docx]

## **Supplementary Material**

# **Engagement and Utilization of the Digital Physical Therapist in Urban and Rural Areas Across the United States**

## **Supplementary table 1:** Primary RUCA Codes[47].

| ***Code*** | ***Description*** |
| --- | --- |
| *1* | Metropolitan area core: Primary flow within an urbanized area (UA) |
| *2* | Metropolitan area high commuting: Primary flow 30% or more to a UA |
| *3* | Micropolitan area core: primary flow within an Urban Cluster of 10,000 to 49,999 (large UC) |
| *4* | Micropolitan high commuting: primary flow 30% or more to a large UC |
| *5* | Micropolitan high commuting: primary flow 30% or more to a large UC |
| *6* | Micropolitan low commuting: primary flow 10% to 30% to a large UC |
| *7* | Small town core: primary flow within an Urban Cluster of 2,500 to 9,999 (small UC) |
| *8* | Small town high commuting: primary flow 30% or more to a small UC |
| *9* | Small town low commuting: primary flow 10% to 30% to a small UC |
| *10* | Rural areas: primary flow to a tract outside a UA or UC |

## **Supplementary table 2:** Baseline characteristics for the patients that completed the 12-week program (Completers), and those that did not (Non-completers).

|  | **Completers (N=7378, 73.8%)** | **Non-Completers**  **(N=2614, 26.2%)** | **P value** |
| --- | --- | --- | --- |
| Demographic Area |  |  | .022 |
| Urban | 6472(87.7) | 2337(89.4) |  |
| Rural | 906(12.3) | 277(10.6) |  |
| Age (years), mean (SD) | 49.38(12.29) | 46.23(12.61) | <.001 |
| Age categories (years), N(%): |  |  | <.001 |
| <25 | 116(1.6) | 68(2.6) |  |
| 25-40 | 1813(24.6) | 883(33.8) |  |
| 40-60 | 3982(54.0) | 1297(49.6) |  |
| >60 | 1467(19.9) | 366(14.0) |  |
| BMI, mean (SD) | 28.81(6.47) | 30.22(7.36) | <.001 |
| BMI categories, N(%): |  |  | <.001 |
| Underweight (<18.5) | 74(1.2) | 16(0.7) |  |
| Normal (18.5-25) | 2170(34.0) | 629(27.3) |  |
| Overweight (25-30) | 1922(30..1) | 657(28.6) |  |
| Obese(30-40) | 1735(27.2) | 733(31.9) |  |
| Obese grade III (>40) | 477(7.5) | 266(11.6) |  |
| Gender, N(%)a: |  |  | 0.991 |
| Woman | 4060(55.0) | 1442(55.2) |  |
| Man | 3294(44.6) | 1163(44.5) |  |
| Nonbinary | 18(0.2) | 6(0.2) |  |
| Prefer not specify | 4(0.1) | 2(0.1) |  |
| Employment Status, N(%)b: |  |  | <.001 |
| Employed full time | 4539(61.5) | 1732(66.3) |  |
| Employed part time | 1781(24.1) | 567(21.7) |  |
| Not employed | 809(11.9) | 258(9.9) |  |
| Education level, N(%): |  |  | <.001 |
| High school or less | 599(8.1) | 267(10.2) |  |
| Some College including bachelor's/Associate's degree | 3283(44.5) | 1260(48.2) |  |
| Some graduate school including Master's/Doctorate degree | 1602(21.7) | 480(18.4) |  |
| Not available / prefers not to answer | 1894(25.7) | 607(23.2) |  |
| Acuity, N(%): |  |  | 0.685 |
| Acute | 1578(21.4) | 569(21.8) |  |
| Chronic | 5800(78.6) | 2045(78.2) |  |
| Anatomical Pain region, N(%): |  |  | 0.010 |
| Ankle | 287(3.9) | 135(5.2) |  |
| Elbow | 220(3.0) | 66(2.5) |  |
| Hip | 682(9.2) | 218(8.3) |  |
| Knee | 1088(14.7) | 350(13.4) |  |
| Low back | 2884(39.1) | 1092(41.8) |  |
| Neck | 688(9.3) | 248(9.5) |  |
| Shoulder | 1225(16.6) | 407(15.6) |  |
| Wrist/hand | 304(4.1) | 98(3.7) |  |
| Clinical Outcomes, (SD) |  |  |  |
| Pain Level | 4.78(1.98) | 4.96(2.02) | 0.616 |
| Surgery Intent>0 | 23.68(23.6) | 25.06(24.63) | 0.095 |
| GAD-7≥5 | 8.85(4.07) | 8.96(4.12) | 0.539 |
| PHQ-9≥5 | 9.14(4.3) | 9.39(4.32) | 0.216 |
| WPAI Overall>0 | 29.38(19.92) | 31.29(20.54) | .004 |
| WPAI Work>0 | 28.08(18.57) | 30.15(19.35) | .001 |
| WPAI Time>0 | 17.8(18.02) | 18.83(18.22) | 0.466 |
| WPAI Activity>0 | 36.99(22.65) | 38.45(23.43) | .014 |

Missing values for completers and non-completers respectively: (a) 2(0.0) and 1(0.0); (b) 249(3.4) and 57(2.2).

##

##

##

##

##

##

##

## **Supplementary table 3:** LGCA models for intention-to-treat analysis.

|  | **Intercept** | | | | | | | | |
| --- | --- | --- | --- | --- | --- | --- | --- | --- | --- |
|  | **Urban** | | | **Rural** | | |  |  |  |
| **Outcome** | **Mean** | **CI** | **p** | **Mean** | **CI** | **p** | **Diff** | **CI** | **p** |
| Pain Level | 4.8 (1.63) | (4.76, 4.84) | < .001 | 4.84 (1.96) | (4.73, 4.96) | < .001 | -0.04 | (-0.16, 0.08) | 0.496 |
| GAD-7≥5 | 8.8 (2.59*)* | (8.35, 9.24) | < .001 | 8.84 (3.18) | (8.67, 9) | < .001 | -0.04 | (-0.51, 0.43) | 0.858 |
| GAD-7 | 3.03 (3.47) | (2.94, 3.12) | < .001 | 2.97 (3.36) | (2.73, 3.22) | < .001 | 0.05 | (-0.21, 0.31) | 0.698 |
| PHQ-9≥5 | 9.31 (4.3) | (8.79, 9.82) | < .001 | 9.2 (4.29) | (9, 9.4) | < .001 | 0.11 | (-0.44, 0.66) | 0.701 |
| PHQ-9 | 2.32 (3.57) | (2.24, 2.41) | < .001 | 2.7 (4.04) | (2.45, 2.95) | < .001 | -0.37 | (-0.64, -0.11) | 0.006 |
| WPAI – WPAI Overall>0 | 29.48 (11.65) | (27.83, 31.12) | < .001 | 29.63 (12.41) | (29.01, 30.25) | < .001 | -0.15 | (-1.91, 1.6) | 0.864 |
| WPAI Overall | 17.12 (14.7) | (16.63, 17.61) | < .001 | 17.85 (13.14) | (16.52, 19.19) | < .001 | -0.73 | (-2.15, 0.69) | 0.311 |
| WPAI – Activity>0 | 36.94 (15.81) | (36.4, 37.49) | < .001 | 37.52 (17.45) | (36.05, 38.98) | < .001 | -0.57 | (-2.13, 0.99) | 0.475 |
| WPAI – Activity | 28.56 (16.68) | (28.03, 29.09) | < .001 | 29.74 (19.73) | (28.29, 31.19) | < .001 | -1.18 | (-2.72, 0.37) | 0.135 |
| WPAI – Work>0 | 28.31 (9.12) | (26.75, 29.88) | < .001 | 28.36 (12.23) | (27.78, 28.95) | < .001 | -0.05 | (-1.72, 1.62) | 0.955 |
| WPAI – Work | 16.04 (13.33) | (15.58, 16.5) | < .001 | 16.92 (12.98) | (15.65, 18.18) | < .001 | -0.88 | (-2.23, 0.47) | 0.202 |
| WPAI - Time Missed>0 | 15.38 (14.85) | (12.5, 18.26) | < .001 | 17.86 (11) | (16.53, 19.19) | < .001 | -2.48 | (-5.65, 0.69) | 0.125 |
| WPAI - Time Missed | 1.92 (4.13) | (1.73, 2.11) | < .001 | 1.65 (6.69) | (1.21, 2.08) | < .001 | 0.27 | (-0.2, 0.75) | 0.26 |

|  | **Slope** | | | | | | | | |
| --- | --- | --- | --- | --- | --- | --- | --- | --- | --- |
|  | **Urban** | | | **Rural** | | |  |  |  |
| **Outcome** | **Mean** | **CI** | **p** | **Mean** | **CI** | **p** | **Diff** | **CI** | **p** |
| Pain Level | -0.44 (0.41) | (-0.45, -0.42) | < .001 | -0.46 (0.51) | (-0.5, -0.42) | < .001 | 0.02 | (-0.03, 0.07) | 0.392 |
| GAD-7≥5 | -0.94 (0.61) | (-1.14, -0.75) | < .001 | -0.95 (0.63) | (-1.01, -0.88) | < .001 | 0.01 | (-0.2, 0.21) | 0.959 |
| GAD-7 | -0.2 (0.41) | (-0.22, -0.17) | < .001 | -0.15 (0.45) | (-0.22, -0.08) | < .001 | -0.05 | (-0.13, 0.03) | 0.195 |
| PHQ-9≥5 | -1.14 (1.06) | (-1.38, -0.91) | < .001 | -1.08 (0.95) | (-1.17, -1) | < .001 | -0.06 | (-0.31, 0.19) | 0.645 |
| PHQ-9 | -0.16 (0.53) | (-0.19, -0.14) | < .001 | -0.25 (0.66) | (-0.32, -0.18) | < .001 | 0.09 | (0.01, 0.16) | 0.027 |
| WPAI – WPAI Overall>0 | -3.28 (1.56) | (-3.89, -2.67) | < .001 | -3.56 (2.44) | (-3.84, -3.28) | < .001 | 0.28 | (-0.4, 0.95) | 0.417 |
| WPAI Overall | -1.51 (1.88) | (-1.7, -1.33) | < .001 | -1.55 (0.98) | (-2, -1.1) | < .001 | 0.03 | (-0.45, 0.52) | 0.891 |
| WPAI – Activity>0 | -4.05 (2.67) | (-4.26, -3.84) | < .001 | -4.05 (2.86) | (-4.6, -3.5) | < .001 | 0 | (-0.59, 0.59) | 0.998 |
| WPAI – Activity | -2.67 (1.79) | (-2.85, -2.49) | < .001 | -2.94 (2.69) | (-3.41, -2.47) | < .001 | 0.27 | (-0.23, 0.77) | 0.298 |
| WPAI – Work>0 | -3.27 (0.89) | (-3.87, -2.67) | < .001 | -3.47 (2.61) | (-3.73, -3.2) | < .001 | 0.2 | (-0.46, 0.86) | 0.55 |
| WPAI – Work | -1.43 (1.48) | (-1.6, -1.26) | < .001 | -1.52 (1.51) | (-1.95, -1.09) | < .001 | 0.09 | (-0.37, 0.55) | 0.715 |
| WPAI - Time Missed>0 | -2.28 (3.46) | (-3.23, -1.32) | < .001 | -3.7 (3.21) | (-4.2, -3.21) | < .001 | 1.43 | (0.35, 2.5) | 0.009 |
| WPAI - Time Missed | -0.12 (0.93) | (-0.2, -0.04) | 0.003 | -0.09 (1.23) | (-0.27, 0.09) | 0.326 | -0.03 | (-0.23, 0.17) | 0.745 |

|  | **Curve** | | | | | | | | |
| --- | --- | --- | --- | --- | --- | --- | --- | --- | --- |
|  | **Urban** | | | **Rural** | | |  |  |  |
| **Outcome** | **Mean** | **CI** | **p** | **Mean** | **CI** | **p** | **Diff** | **CI** | **p** |
| Pain Level | 0.02 (0.03) | (0.02, 0.02) | < .001 | 0.02 (0.03) | (0.02, 0.03) | < .001 | 0 | (-0.01, 0) | 0.505 |
| GAD-7≥5 | 0.05 (0.03) | (0.03, 0.06) | < .001 | 0.05 (0.04) | (0.04, 0.05) | < .001 | 0 | (-0.02, 0.02) | 0.985 |
| GAD-7 | 0.01 (0.03) | (0.01, 0.01) | < .001 | 0 (0.03) | (0, 0.01) | 0.149 | 0 | (0, 0.01) | 0.271 |
| PHQ-9≥5 | 0.06 (0.05) | (0.04, 0.08) | < .001 | 0.06 (0.06) | (0.05, 0.06) | < .001 | 0.01 | (-0.01, 0.03) | 0.491 |
| PHQ-9 | 0.01 (0.03) | (0.01, 0.01) | < .001 | 0.01 (0.03) | (0.01, 0.02) | < .001 | -0.01 | (-0.01, 0) | 0.077 |
| WPAI – WPAI Overall>0 | 0.17 (0.03) | (0.12, 0.23) | < .001 | 0.19 (0.12) | (0.16, 0.21) | < .001 | -0.02 | (-0.07, 0.04) | 0.59 |
| WPAI Overall | 0.08 (0.1) | (0.06, 0.09) | < .001 | 0.08 (0.03) | (0.04, 0.12) | < .001 | 0 | (-0.04, 0.04) | 0.846 |
| WPAI – Activity>0 | 0.2 (0.12) | (0.18, 0.22) | < .001 | 0.21 (0.03) | (0.16, 0.26) | < .001 | -0.01 | (-0.06, 0.04) | 0.778 |
| WPAI – Activity | 0.13 (0.13) | (0.11, 0.14) | < .001 | 0.15 (0.03) | (0.11, 0.19) | < .001 | -0.02 | (-0.06, 0.02) | 0.276 |
| WPAI – Work>0 | 0.17 (0.03) | (0.12, 0.23) | < .001 | 0.18 (0.15) | (0.16, 0.21) | < .001 | -0.01 | (-0.07, 0.05) | 0.758 |
| WPAI – Work | 0.07 (0.09) | (0.06, 0.08) | < .001 | 0.08 (0.03) | (0.04, 0.12) | < .001 | -0.01 | (-0.05, 0.03) | 0.639 |
| WPAI - Time Missed>0 | 0.11 (0.03) | (0.04, 0.18) | 0.002 | 0.23 (0.03) | (0.18, 0.27) | < .001 | -0.12 | (-0.2, -0.03) | 0.005 |
| WPAI - Time Missed | 0.01 (0.03) | (0, 0.01) | 0.107 | 0 (0.03) | (-0.01, 0.02) | 0.552 | 0 | (-0.02, 0.02) | 0.899 |

## **Supplementary table 4:** Model fit for both filtered and unfiltered models considering intention-to-treat analysis.

|  |  | **Model Fit** | |  |  |
| --- | --- | --- | --- | --- | --- |
|  | **Fit** | | | | |
| **Outcome** | **Chi-sq (df)** | ***p*** | **RMSEA** | **CFI** | **SRMR** |
| Pain Level | 297.06 (2) | < .001 | 0.172 | 0.94 | 0.043 |
| GAD-7≥5 | 56.46 (2) | < .001 | 0.141 | 0.96 | 0.036 |
| GAD-7 | 14.4 (2) | < .001 | 0.035 | 1 | 0.007 |
| PHQ-9≥5 | 29.81 (2) | < .001 | 0.116 | 0.98 | 0.03 |
| PHQ-9 | 7.9 (2) | 0.019 | 0.024 | 1 | 0.005 |
| WPAI – WPAI Overall>0 | 59.33 (2) | < .001 | 0.112 | 0.96 | 0.035 |
| WPAI Overall | 27.11 (2) | < .001 | 0.055 | 0.99 | 0.017 |
| WPAI – Activity>0 | 142.39 (2) | < .001 | 0.135 | 0.96 | 0.036 |
| WPAI – Activity | 74.74 (2) | < .001 | 0.085 | 0.98 | 0.022 |
| WPAI – Work>0 | 69.87 (2) | < .001 | 0.123 | 0.95 | 0.038 |
| WPAI – Work | 28.55 (2) | < .001 | 0.056 | 0.99 | 0.017 |
| WPAI - Time Missed>0 | 26.85 (2) | < .001 | 0.173 | 0.76 | 0.075 |
| WPAI - Time Missed | 3.2 (2) | 0.202 | 0.012 | 1 | 0.007 |

Model appropriateness was assessed using chi-squared test, standardized root mean square residual (SRMR), root mean square error approximation (RMSEA), and confirmatory fit index (CFI), applying the following cut-off criteria: CFI = close to 0.95; RMSEA = close to 0.06 and SRMR = close to 0.08[60, 61].

## **Supplementary table 5:** Conditional Growth-mixture modeling analysis: intent-to-treat for filtered cases.

|  |  | ***Age*** | | | | | | | | |
| --- | --- | --- | --- | --- | --- | --- | --- | --- | --- | --- |
|  |  | Intercept | 95% - CI | *P* | Slope | 95% - CI | *P* | Curve | 95% - CI | *P* |
| Pain | Urban | 0 | (0, 0.01) | ***0.049*** | 0 | (0, 0) | 0.979 | 0 | (0, 0) | 0.94 |
|  | Rural | 0 | (0, 0.01) | 0.350 | 0 | (0, 0) | 0.282 | 0 | (0, 0.01) | 0.410 |
|  | Difference | 0 | (-0.01, 0.01) | 0.864 | 0 | (0, 0) | 0.318 | 0 | (-0.01, 0) | 0.424 |
| GAD-7 | Urban | -0.03 | (-0.06, 0.01) | 0.153 | 0 | (0, 0) | 0.178 | 0.01 | (0, 0.03) | 0.111 |
|  | Rural | -0.02 | (-0.03, 0) | ***0.029*** | 0 | (0, 0) | 0.063 | 0.01 | (0, 0.01) | 0.081 |
|  | Difference | -0.01 | (-0.05, 0.03) | 0.576 | 0 | (0, 0) | 0.504 | 0.01 | (-0.01, 0.02) | 0.385 |
| PHQ-9 | Urban | -0.01 | (-0.06, 0.03) | 0.548 | 0 | (0, 0) | 0.691 | 0 | (-0.01, 0.02) | 0.684 |
|  | Rural | -0.01 | (-0.03, 0) | 0.103 | 0 | (0, 0) | 0.979 | 0 | (-0.01, 0.01) | 0.751 |
|  | Difference | 0 | (-0.05, 0.05) | 0.978 | 0 | (0, 0) | 0.704 | 0 | (-0.02, 0.02) | 0.799 |
| WPAI Overall | Urban | -0.02 | (-0.18, 0.13) | 0.754 | 0 | (-0.01, 0) | 0.605 | 0.02 | (-0.04, 0.07) | 0.567 |
|  | Rural | -0.05 | (-0.11, 0) | 0.056 | 0 | (0, 0) | 0.226 | -0.01 | (-0.03, 0.02) | 0.537 |
|  | Difference | 0.03 | (-0.14, 0.19) | 0.742 | 0 | (-0.01, 0) | 0.334 | 0.02 | (-0.04, 0.08) | 0.445 |
| WPAI Activity | Urban | -0.03 | (-0.08, 0.02) | 0.187 | 0 | (0, 0) | 0.715 | 0 | (-0.02, 0.02) | 0.81 |
|  | Rural | 0.06 | (-0.07, 0.18) | 0.361 | 0 | (0, 0) | 0.994 | 0 | (-0.05, 0.05) | 0.982 |
|  | Difference | -0.09 | (-0.22, 0.04) | 0.187 | 0 | (0, 0) | 0.898 | 0 | (-0.05, 0.05) | 0.949 |
| WPAI Work | Urban | -0.01 | (-0.16, 0.14) | 0.851 | 0 | (-0.01, 0) | 0.5 | 0.02 | (-0.03, 0.07) | 0.473 |
|  | Rural | -0.06 | (-0.11, -0.01) | ***0.027*** | 0 | (0, 0) | 0.242 | -0.01 | (-0.03, 0.02) | 0.622 |
|  | Difference | 0.04 | (-0.12, 0.2) | 0.595 | 0 | (-0.01, 0) | 0.278 | 0.03 | (-0.03, 0.08) | 0.395 |
| WPAI Time | Urban | 0.01 | (-0.29, 0.3) | 0.97 | 0 | (-0.01, 0) | 0.406 | 0.02 | (-0.07, 0.11) | 0.645 |
|  | Rural | -0.02 | (-0.13, 0.08) | 0.668 | 0 | (0, 0) | 0.969 | 0 | (-0.04, 0.03) | 0.828 |
|  | Difference | 0.03 | (-0.28, 0.34) | 0.856 | 0 | (-0.01, 0) | 0.443 | 0.03 | (-0.07, 0.12) | 0.611 |

|  |  | ***Gender*** | | | | | | | | |
| --- | --- | --- | --- | --- | --- | --- | --- | --- | --- | --- |
|  |  | Intercept | 95% - CI | *P* | Slope | 95% - CI | *P* | Curve | 95% - CI | *P* |
| Pain | Urban | 0.16 | (0.07, 0.24) | ***< .001*** | 0 | (0, 0) | 0.619 | -0.01 | (-0.04,0.03) | 0.715 |
|  | Rural | 0.17 | (-0.06,0.41) | 0.149 | 0 | (0, 0.01) | 0.254 | -0.06 | (-0.14,0.03) | 0.206 |
|  | Difference | -0.01 | (-0.26,0.23) | 0.911 | 0 | (-0.01, 0) | 0.374 | 0.05 | (-0.04,0.14) | 0.289 |
| GAD-7 | Urban | 0.11 | (-0.8, 1.02) | 0.810 | 0.03 | (0, 0.06) | 0.090 | -0.23 | (-0.61,0.15) | 0.231 |
|  | Rural | 0.19 | (-0.14,0.51) | 0.271 | 0 | (-0.01,0.01) | 0.586 | -0.03 | (-0.16, 0.1) | 0.644 |
|  | Difference | -0.07 | (-1.04, 0.9) | 0.883 | 0.03 | (-0.01,0.06) | 0.15 | -0.2 | (-0.6, 0.2) | 0.328 |
| PHQ-9 | Urban | 0.1 | (-0.92,1.12) | 0.846 | 0.04 | (0, 0.08) | 0.078 | -0.43 | (-0.9, 0.04) | 0.074 |
|  | Rural | -0.03 | (-0.44,0.37) | 0.871 | -0.01 | (-0.02,0.01) | 0.477 | 0.06 | (-0.12,0.23) | 0.524 |
|  | Difference | 0.13 | (-0.96,1.23) | 0.810 | 0.04 | (0, 0.08) | 0.058 | -0.48 | (-0.99,0.02) | 0.058 |
| WPAI Overall | Urban | 1.71 | (-1.77,5.19) | 0.335 | 0 | (-0.11, 0.1) | 0.937 | -0.06 | (-1.31,1.19) | 0.927 |
|  | Rural | 1.09 | (-0.17,2.35) | 0.090 | 0.01 | (-0.03,0.06) | 0.646 | -0.13 | (-0.67,0.41) | 0.645 |
|  | Difference | 0.62 | (-3.08,4.33) | 0.741 | -0.01 | (-0.13, 0.1) | 0.799 | 0.07 | (-1.29,1.43) | 0.921 |
| WPAI Activity | Urban | 0.38 | (-0.73,1.48) | 0.506 | -0.02 | (-0.05,0.01) | 0.258 | 0.19 | (-0.23,0.61) | 0.377 |
|  | Rural | -0.9 | (-3.94,2.14) | 0.562 | -0.01 | (-0.1, 0.09) | 0.892 | 0.15 | (-1.01,1.31) | 0.800 |
|  | Difference | 1.28 | (-1.96,4.51) | 0.44 | -0.01 | (-0.11,0.09) | 0.797 | 0.04 | (-1.19,1.27) | 0.951 |
| WPAI Work | Urban | 1.16 | (-2.15,4.47) | 0.492 | 0.01 | (-0.09,0.11) | 0.873 | -0.06 | (-1.27,1.15) | 0.924 |
|  | Rural | 0.87 | (-0.32,2.05) | 0.152 | 0.01 | (-0.03,0.05) | 0.680 | -0.13 | (-0.64,0.39) | 0.625 |
|  | Difference | 0.29 | (-3.22,3.81) | 0.869 | 0 | (-0.11,0.11) | 0.992 | 0.07 | (-1.25,1.38) | 0.918 |
| WPAI Time | Urban | 1.81 | (-4.44,8.05) | 0.571 | 0.02 | (-0.1, 0.14) | 0.744 | -0.57 | (-2.35,1.22) | 0.534 |
|  | Rural | -1.87 | (-4.61,0.87) | 0.180 | 0 | (-0.07,0.08) | 0.990 | 0.29 | (-0.72,1.31) | 0.570 |
|  | Difference | 3.68 | (-3.14,10.5) | 0.290 | 0.02 | (-0.12,0.16) | 0.787 | -0.86 | (-2.92,1.19) | 0.411 |

|  |  | ***BMI*** | | | | | | | | |
| --- | --- | --- | --- | --- | --- | --- | --- | --- | --- | --- |
|  |  | Intercept | 95% - CI | *P* | Slope | 95% - CI | *P* | Curve | 95% - CI | *P* |
| Pain | Urban | 0.01 | (0.01, 0.02) | ***< .001*** | 0 | (0, 0) | 0.258 | 0 | (0, 0) | 0.284 |
|  | Rural | 0.01 | (0, 0.03) | 0.122 | 0 | (0, 0) | 0.471 | 0 | (0, 0.01) | 0.307 |
|  | Difference | 0 | (-0.02,0.02) | 0.953 | 0 | (0, 0) | 0.796 | 0 | (-0.01, 0) | 0.596 |
| GAD-7 | Urban | 0.01 | (-0.06,0.07) | 0.834 | 0 | (0, 0) | 0.653 | -0.01 | (-0.03,0.01) | 0.452 |
|  | Rural | 0.02 | (-0.01,0.04) | 0.165 | 0 | (0, 0) | 0.395 | 0 | (-0.01,0.01) | 0.596 |
|  | Difference | -0.01 | (-0.08,0.06) | 0.755 | 0 | (0, 0) | 0.936 | -0.01 | (-0.03,0.02) | 0.636 |
| PHQ-9 | Urban | 0.04 | (-0.03, 0.1) | 0.277 | 0 | (0, 0) | 0.852 | -0.01 | (-0.04,0.02) | 0.591 |
|  | Rural | 0.02 | (0, 0.05) | 0.104 | 0 | (0, 0) | 0.298 | -0.01 | (-0.02,0.01) | 0.26 |
|  | Difference | 0.01 | (-0.06,0.09) | 0.705 | 0 | (0, 0) | 0.87 | 0 | (-0.03,0.03) | 0.931 |
| WPAI Overall | Urban | 0.3 | (0.08, 0.52) | ***0.007*** | 0 | (-0.01,0.01) | 0.97 | -0.04 | (-0.13,0.05) | 0.353 |
|  | Rural | 0.09 | (0, 0.18) | 0.059 | 0 | (0, 0) | 0.975 | 0 | (-0.04,0.04) | 0.876 |
|  | Difference | 0.21 | (-0.03,0.45) | 0.084 | 0 | (-0.01,0.01) | 0.983 | -0.05 | (-0.14,0.05) | 0.36 |
| WPAI Activity | Urban | 0.18 | (0.1, 0.27) | ***< .001*** | 0 | (0, 0) | 0.938 | 0 | (-0.04,0.03) | 0.843 |
|  | Rural | 0.37 | (0.17, 0.57) | ***< .001*** | 0 | (-0.01,0.01) | 0.848 | -0.01 | (-0.09,0.07) | 0.747 |
|  | Difference | -0.18 | (-0.4, 0.04) | 0.102 | 0 | (-0.01,0.01) | 0.881 | 0.01 | (-0.07,0.09) | 0.822 |
| WPAI Work | Urban | 0.26 | (0.05, 0.46) | ***0.014*** | 0 | (-0.01,0.01) | 0.989 | -0.04 | (-0.13,0.05) | 0.346 |
|  | Rural | 0.09 | (0, 0.17) | 0.057 | 0 | (0, 0) | 0.702 | 0.01 | (-0.03,0.04) | 0.671 |
|  | Difference | 0.17 | (-0.05, 0.4) | 0.129 | 0 | (-0.01,0.01) | 0.869 | -0.05 | (-0.14,0.04) | 0.301 |
| WPAI Time | Urban | -0.05 | (-0.3, 0.21) | 0.728 | -0.01 | (-0.02, 0) | ***0.023*** | 0.13 | (0.02, 0.25) | ***0.025*** |
|  | Rural | 0.05 | (-0.13,0.23) | 0.574 | 0 | (-0.01, 0) | 0.944 | -0.01 | (-0.08,0.06) | 0.810 |
|  | Difference | -0.1 | (-0.41,0.22) | 0.543 | -0.01 | (-0.02, 0) | 0.053 | 0.14 | (0.01, 0.28) | ***0.041*** |

|  |  | ***Employed*** | | | | | | | | |
| --- | --- | --- | --- | --- | --- | --- | --- | --- | --- | --- |
|  |  | Intercept | 95% - CI | *P* | Slope | 95% - CI | *P* | Curve | 95% - CI | *P* |
| Pain | Urban | -0.12 | (-0.28, 0.03) | 0.105 | 0 | (-0.01, 0) | 0.482 | 0.01 | (-0.04,0.07) | 0.651 |
|  | Rural | -0.05 | (-0.37, 0.27) | 0.763 | 0 | (-0.01, 0.01) | 0.967 | -0.01 | (-0.13,0.11) | 0.868 |
|  | Difference | -0.07 | (-0.43, 0.28) | 0.681 | 0 | (-0.01, 0.01) | 0.732 | 0.02 | (-0.11,0.16) | 0.733 |
| GAD-7 | Urban | -0.08 | (-1.24, 1.08) | 0.893 | -0.78 | (-1.41, -0.16) | ***0.014*** | 0.7 | (-0.62,2.03) | 0.296 |
|  | Rural | 0 | (-0.04, 0.04) | 0.869 | -0.01 | (-0.03, 0.01) | 0.529 | 0 | (-0.04,0.05) | 0.902 |
|  | Difference | 0.04 | (-0.43, 0.5) | 0.879 | 0.05 | (-0.19, 0.29) | 0.676 | -0.01 | (-0.54,0.51) | 0.956 |
| PHQ-9 | Urban | -0.52 | (-2.02, 0.98) | 0.499 | -0.05 | (-0.1, -0.01) | ***0.026*** | 0.63 | (0.08, 1.17) | ***0.024*** |
|  | Rural | -1.27 | (-2.06,-0.47) | ***0.002*** | -0.02 | (-0.04, 0.01) | 0.300 | 0.23 | (-0.1, 0.55) | 0.174 |
|  | Difference | 0.75 | (-0.95, 2.44) | 0.387 | -0.04 | (-0.09, 0.02) | 0.175 | 0.4 | (-0.23,1.04) | 0.215 |
| WPAI Overall | Urban | -2.47 | (-7.63, 2.68) | 0.347 | -0.04 | (-0.2, 0.12) | 0.637 | 0.82 | (-1.23,2.86) | 0.433 |
|  | Rural | -1.53 | (-4.35, 1.29) | 0.288 | 0.05 | (-0.04, 0.13) | 0.306 | -0.33 | (-1.39,0.74) | 0.548 |
|  | Difference | -0.95 | (-6.83, 4.93) | 0.752 | -0.08 | (-0.27, 0.1) | 0.363 | 1.15 | (-1.16,3.45) | 0.331 |
| WPAI Activity | Urban | -3.76 | (-5.76,-1.75) | ***< .001*** | -0.04 | (-0.1, 0.02) | 0.183 | 0.34 | (-0.39,1.07) | 0.357 |
|  | Rural | -1.93 | (-6.04, 2.19) | 0.358 | 0 | (-0.13, 0.12) | 0.948 | 0.14 | (-1.33, 1.6) | 0.855 |
|  | Difference | -1.83 | (-6.41, 2.75) | 0.434 | -0.04 | (-0.17, 0.1) | 0.598 | 0.21 | (-1.43,1.84) | 0.804 |
| WPAI Work | Urban | -2.31 | (-7.42, 2.81) | 0.377 | -0.07 | (-0.23, 0.08) | 0.335 | 1.18 | (-0.76,3.13) | 0.232 |
|  | Rural | -1.76 | (-4.42, 0.91) | 0.197 | 0.02 | (-0.06, 0.1) | 0.699 | -0.04 | (-1.06,0.97) | 0.931 |
|  | Difference | -0.55 | (-6.31, 5.22) | 0.852 | -0.09 | (-0.26, 0.08) | 0.302 | 1.23 | (-0.96,3.42) | 0.272 |
| WPAI Time | Urban | 5.73 | (-0.83,12.28) | 0.087 | 0.12 | (-0.04, 0.28) | 0.155 | -1.38 | (-3.45,0.68) | 0.189 |
|  | Rural | -3.24 | (-8.74, 2.27) | 0.250 | 0.1 | (-0.1, 0.3) | 0.342 | -0.81 | (-3.57,1.94) | 0.563 |
|  | Difference | 8.96 | (0.4, 17.52) | ***0.040*** | 0.02 | (-0.24, 0.28) | 0.890 | -0.57 | (-4.02,2.88) | 0.745 |

|  |  | ***Chronic Pain*** | | | | | | | | |
| --- | --- | --- | --- | --- | --- | --- | --- | --- | --- | --- |
|  |  | Intercept | 95% - CI | *P* | Slope | 95% - CI | *P* | Curve | 95% - CI | *P* |
| Pain | Urban | 0.03 | (-0.08, 0.13) | 0.618 | 0 | (-0.01, 0) | ***0.005*** | 0.09 | (0.05, 0.13) | ***< .001*** |
|  | Rural | 0.22 | (-0.08, 0.53) | 0.145 | 0 | (-0.01, 0.01) | 0.629 | 0.06 | (-0.05, 0.17) | 0.324 |
|  | Difference | -0.2 | (-0.52, 0.12) | 0.223 | 0 | (-0.01, 0.01) | 0.601 | 0.04 | (-0.08, 0.15) | 0.527 |
| GAD-7 | Urban | 0.4 | (-1.04, 1.83) | 0.590 | 0 | (-0.05, 0.04) | 0.819 | 0.17 | (-0.32, 0.65) | 0.498 |
|  | Rural | 0.24 | (-0.16, 0.64) | 0.237 | -0.01 | (-0.02, 0) | 0.206 | 0.14 | (-0.03, 0.31) | 0.103 |
|  | Difference | 0.16 | (-1.34, 1.65) | 0.839 | 0 | (-0.04, 0.05) | 0.859 | 0.03 | (-0.49, 0.54) | 0.915 |
| PHQ-9 | Urban | -0.54 | (-2.46, 1.38) | 0.580 | -0.06 | (-0.11, -0.01) | ***0.013*** | 0.89 | (0.32, 1.45) | ***0.002*** |
|  | Rural | 0.4 | (-0.08, 0.87) | 0.100 | 0.02 | (0, 0.03) | 0.117 | -0.17 | (-0.39, 0.05) | 0.137 |
|  | Difference | -0.94 | (-2.92, 1.04) | 0.351 | -0.07 | (-0.13, -0.02) | ***0.004*** | 1.06 | (0.45, 1.66) | ***< .001*** |
| WPAI Overall | Urban | -1.04 | (-5.67, 3.6) | 0.661 | -0.06 | (-0.21, 0.1) | 0.466 | 0.85 | (-0.94, 2.63) | 0.352 |
|  | Rural | -0.79 | (-2.37, 0.78) | 0.324 | -0.09 | (-0.15, -0.04) | ***0.001*** | 1.22 | (0.56, 1.89) | ***< .001*** |
|  | Difference | -0.24 | (-5.14, 4.65) | 0.923 | 0.04 | (-0.13, 0.2) | 0.669 | -0.38 | (-2.28, 1.52) | 0.696 |
| WPAI Activity | Urban | -1.21 | (-2.56, 0.15) | 0***.***080 | -0.09 | (-0.13, -0.05) | ***< .001*** | 1.4 | (0.89, 1.9) | ***< .001*** |
|  | Rural | 0.37 | (-3.63, 4.36) | 0.858 | -0.03 | (-0.15, 0.09) | 0.619 | 0.77 | (-0.73, 2.26) | 0.316 |
|  | Difference | -1.57 | (-5.8, 2.65) | 0.465 | -0.06 | (-0.19, 0.06) | 0.325 | 0.63 | (-0.95, 2.21) | 0.433 |
| WPAI Work | Urban | -0.48 | (-4.89, 3.93) | 0.831 | -0.07 | (-0.21, 0.08) | 0.374 | 0.77 | (-0.93, 2.48) | 0.373 |
|  | Rural | 0.1 | (-1.37, 1.56) | 0.898 | -0.06 | (-0.11, -0.01) | 0.021 | 0.82 | (0.19, 1.44) | ***0.011*** |
|  | Difference | -0.58 | (-5.23, 4.07) | 0.808 | -0.01 | (-0.16, 0.15) | 0.938 | -0.04 | (-1.86, 1.77) | 0.965 |
| WPAI Time | Urban | -1.89 | (-9.81, 6.03) | 0.640 | 0.02 | (-0.11, 0.15) | 0.754 | -0.25 | (-2.48, 1.98) | 0.828 |
|  | Rural | -5.41 | (-8.93, -1.9) | ***0.003*** | -0.14 | (-0.23, -0.06) | ***< .001*** | 2.18 | (1.06, 3.31) | ***< .001*** |
|  | Difference | 3.52 | (-5.14, 12.19) | 0.425 | 0.16 | (0.01, 0.31) | ***0.032*** | -2.43 | (-4.93, 0.07) | 0.056 |

##

## **Supplementary table 6**: Mean changes between baseline and program-end and mean differences between groups for the studied clinical outcomes, following an per-protocol analysis.

|  | **Urban** | | **Rural** | | **Mean Difference** | |
| --- | --- | --- | --- | --- | --- | --- |
| **Outcome** | **Mean change (95% CI)** | ***P*** | **Mean change**  **(95% CI)** | ***P*** | **Diff (95% CI)** | ***P*** |
| *Pain Level* | 2.2  (2.1, 2.3) | < .001 | 2.2  (2.0, 2.4) | < .001 | 0  (-0.2, 0.2) | 0.995 |
| *GAD-7* | 1.24  (1.13, 1.35) | < .001 | 1.04  (0.74, 1.35) | < .001 | 0.2  (-0.12, 0.52) | 0.228 |
| *GAD-7≥5* | 4.2  (3.3, 5.0) | < .001 | 4.7  (4.4, 5.0) | < .001 | -0.5  (-1.4, 0.4) | 0.250 |
| *PHQ-9* | 0.9  (0.79, 1) | < .001 | 1.06  (0.76, 1.36) | < .001 | -0.16  (-0.48, 0.15) | 0.308 |
| *PHQ-9≥5* | 4.3  (3.2, 5.3) | < .001 | 5.0  (4.6, 5.3) | < .001 | -0.7  (-1.8, 0.4) | 0.210 |
| *WPAI - Overall* | 7.31  (6.58, 8.05) | < .001 | 7.27  (5.29, 9.25) | < .001 | 0.04  (-2.07, 2.15) | 0.971 |
| *WPAI - Overall>0* | 14.4  (11.5, 17.2) | < .001 | 15.7  (14.6, 16.8) | < .001 | -1.3  (-4.4, 1.7) | 0.394 |
| *WPAI - Work* | 13.69  (12.92, 14.46) | < .001 | 12.96  (11.01, 14.9) | < .001 | 0.73  (-1.36, 2.82) | 0.492 |
| *WPAI -*  *Work>0* | 19.4  (18.5, 20.3) | < .001 | 17.6  (15.3, 19.9) | < .001 | 1.8  (-0.7, 4.3) | 0.154 |
| *WPAI - Time Missed* | 7.08  (6.39, 7.77) | < .001 | 6.96  (5.07, 8.84) | < .001 | 0.13  (-1.88, 2.13) | 0.902 |
| *WPAI -*  *Time Missed >0* | 14.2  (11.5, 17.00) | < .001 | 15.4  (14.3, 16.5) | < .001 | -1.2  (-4.1, 1.8) | 0.435 |
| *WPAI - Activity* | 0.68  (0.38, 0.97) | < .001 | 0.24  (-0.58, 1.05) | 0.568 | 0.44  (-0.43, 1.3) | 0.321 |
| *WPAI -*  *Activity >0* | 10.6  (4.2, 17.1) | 0.001 | 12.2  (10.5, 13.9) | < .001 | -1.6  (-8.3, 5.1) | 0.643 |

*Abbreviations:* CI denotes confidence intervals. GAD-7, Generalized Anxiety Disorder 7-item scale; PHQ-9, Patient Health 9-item questionnaire; WPAI, Work Productivity and Activity Impairment questionnaire

##

## **Supplementary table 7:** LGCA models for per-protocol analysis.

|  | **Intercept** | | | | | | | | |
| --- | --- | --- | --- | --- | --- | --- | --- | --- | --- |
|  | **Urban** | | | **Rural** | | |  |  |  |
| **Outcome** | **Mean** | **CI** | **p** | **Mean** | **CI** | **p** | **Diff** | **CI** | **p** |
| Pain Level | 4.76 (1.63) | (4.71, 4.81) | < .001 | 4.74 (1.97) | (4.61, 4.87) | < .001 | 0.02 | (-0.12, 0.15) | 0.809 |
| GAD-7≥5 | 8.48 (2.27) | (8.01, 8.96) | < .001 | 8.83 (3.1) | (8.64, 9.03) | < .001 | -0.35 | (-0.86, 0.17) | 0.185 |
| GAD-7 | 2.91 (3.46) | (2.81, 3.02) | < .001 | 2.83 (3.11) | (2.57, 3.1) | < .001 | 0.08 | (-0.2, 0.36) | 0.579 |
| PHQ-9≥5 | 8.89 (3.63) | (8.33, 9.46) | < .001 | 9.17 (4.31) | (8.93, 9.41) | < .001 | -0.28 | (-0.89, 0.33) | 0.371 |
| PHQ-9 | 2.2 (3.56) | (2.1, 2.3) | < .001 | 2.51 (3.65) | (2.25, 2.78) | < .001 | -0.31 | (-0.6, -0.03) | 0.031 |
| WPAI – WPAI Overall>0 | 28.38 (13.61) | (26.53, 30.24) | < .001 | 29.19 (13.38) | (28.47, 29.9) | < .001 | -0.8 | (-2.79, 1.18) | 0.429 |
| WPAI Overall | 16.67 (14.92) | (16.11, 17.24) | < .001 | 17.47 (14.22) | (15.98, 18.96) | < .001 | -0.8 | (-2.39, 0.79) | 0.325 |
| WPAI – Activity>0 | 36.52 (15.41) | (35.88, 37.15) | < .001 | 36.6 (15.97) | (34.97, 38.22) | < .001 | -0.08 | (-1.82, 1.66) | 0.93 |
| WPAI – Activity | 28.26 (16.45) | (27.64, 28.87) | < .001 | 28.91 (18.11) | (27.3, 30.52) | < .001 | -0.65 | (-2.38, 1.07) | 0.457 |
| WPAI – Work>0 | 27.47 (13.15) | (25.71, 29.24) | < .001 | 27.85 (12.87) | (27.18, 28.53) | < .001 | -0.38 | (-2.27, 1.51) | 0.695 |
| WPAI – Work | 15.6 (13.77) | (15.07, 16.13) | < .001 | 16.59 (13.63) | (15.18, 18.01) | < .001 | -0.99 | (-2.51, 0.52) | 0.198 |
| WPAI - Time Missed>0 | 15 (14.67) | (11.63, 18.36) | < .001 | 17.23 (7.87) | (15.7, 18.76) | < .001 | -2.23 | (-5.92, 1.47) | 0.237 |
| WPAI - Time Missed | 1.87 (4.07) | (1.65, 2.08) | < .001 | 1.5 (6.48) | (1.04, 1.97) | < .001 | 0.36 | (-0.15, 0.88) | 0.168 |

|  | **Slope** | | | | | | | | |
| --- | --- | --- | --- | --- | --- | --- | --- | --- | --- |
|  | **Urban** | | | **Rural** | | |  |  |  |
| **Outcome** | **Mean** | **CI** | **p** | **Mean** | **CI** | **p** | **Diff** | **CI** | **p** |
| Pain Level | -0.44 (0.41) | (-0.45, -0.42) | < .001 | -0.45 (0.5) | (-0.5, -0.41) | < .001 | 0.02 | (-0.03, 0.07) | 0.494 |
| GAD-7≥5 | -0.94 (0.52) | (-1.14, -0.75) | < .001 | -0.95 (0.58) | (-1.02, -0.88) | < .001 | 0 | (-0.21, 0.21) | 0.981 |
| GAD-7 | -0.2 (0.41) | (-0.22, -0.17) | < .001 | -0.14 (0.42) | (-0.22, -0.07) | < .001 | -0.05 | (-0.13, 0.02) | 0.174 |
| PHQ-9≥5 | -1.15 (0.88) | (-1.39, -0.92) | < .001 | -1.06 (0.96) | (-1.16, -0.97) | < .001 | -0.09 | (-0.34, 0.17) | 0.497 |
| PHQ-9 | -0.15 (0.55) | (-0.18, -0.13) | < .001 | -0.24 (0.57) | (-0.31, -0.17) | < .001 | 0.09 | (0.01, 0.16) | 0.026 |
| WPAI – WPAI Overall>0 | -3.38 (2.1) | (-4.02, -2.74) | < .001 | -3.57 (2.83) | (-3.87, -3.27) | < .001 | 0.19 | (-0.52, 0.89) | 0.601 |
| WPAI Overall | -1.52 (2.22) | (-1.71, -1.32) | < .001 | -1.74 (1.47) | (-2.2, -1.27) | < .001 | 0.22 | (-0.28, 0.72) | 0.387 |
| WPAI – Activity>0 | -3.96 (2.61) | (-4.19, -3.73) | < .001 | -4.19 (2.7) | (-4.78, -3.6) | < .001 | 0.23 | (-0.41, 0.86) | 0.48 |
| WPAI – Activity | -2.64 (1.8) | (-2.84, -2.45) | < .001 | -3.04 (2.42) | (-3.55, -2.54) | < .001 | 0.4 | (-0.14, 0.94) | 0.148 |
| WPAI – Work>0 | -3.44 (2.22) | (-4.06, -2.82) | < .001 | -3.42 (2.93) | (-3.71, -3.14) | < .001 | -0.02 | (-0.7, 0.67) | 0.958 |
| WPAI – Work | -1.42 (2) | (-1.6, -1.24) | < .001 | -1.74 (1.64) | (-2.19, -1.3) | < .001 | 0.32 | (-0.15, 0.8) | 0.183 |
| WPAI - Time Missed>0 | -2.09 (3.3) | (-3.22, -0.95) | < .001 | -3.41 (2.31) | (-3.91, -2.91) | < .001 | 1.32 | (0.08, 2.56) | 0.037 |
| WPAI - Time Missed | -0.14 (0.89) | (-0.23, -0.05) | 0.002 | -0.04 (1.09) | (-0.23, 0.14) | 0.645 | -0.1 | (-0.3, 0.11) | 0.348 |

|  | **Curve** | | | | | | | | |
| --- | --- | --- | --- | --- | --- | --- | --- | --- | --- |
|  | **Urban** | | | **Rural** | | |  |  |  |
| **Outcome** | **Mean** | **CI** | **p** | **Mean** | **CI** | **p** | **Diff** | **CI** | **p** |
| Pain Level | 0.02 (0.03) | (0.02, 0.02) | < .001 | 0.02 (0.03) | (0.02, 0.03) | < .001 | 0 | (-0.01, 0) | 0.499 |
| GAD-7≥5 | 0.05 (0.03) | (0.03, 0.07) | < .001 | 0.05 (0.04) | (0.04, 0.05) | < .001 | 0 | (-0.01, 0.02) | 0.709 |
| GAD-7 | 0.01 (0.03) | (0.01, 0.01) | < .001 | 0 (0.03) | (0, 0.01) | 0.142 | 0 | (0, 0.01) | 0.325 |
| PHQ-9≥5 | 0.07 (0.05) | (0.05, 0.09) | < .001 | 0.05 (0.06) | (0.05, 0.06) | < .001 | 0.01 | (-0.01, 0.03) | 0.277 |
| PHQ-9 | 0.01 (0.03) | (0, 0.01) | < .001 | 0.01 (0.03) | (0.01, 0.02) | < .001 | -0.01 | (-0.01, 0) | 0.053 |
| WPAI – WPAI Overall>0 | 0.18 (0.03) | (0.13, 0.24) | < .001 | 0.19 (0.14) | (0.16, 0.21) | < .001 | -0.01 | (-0.07, 0.05) | 0.834 |
| WPAI Overall | 0.08 (0.12) | (0.06, 0.09) | < .001 | 0.09 (0.03) | (0.06, 0.13) | < .001 | -0.02 | (-0.06, 0.02) | 0.382 |
| WPAI – Activity>0 | 0.2 (0.13) | (0.18, 0.21) | < .001 | 0.23 (0.04) | (0.18, 0.28) | < .001 | -0.03 | (-0.09, 0.02) | 0.25 |
| WPAI – Activity | 0.13 (0.14) | (0.11, 0.14) | < .001 | 0.16 (0.03) | (0.12, 0.2) | < .001 | -0.04 | (-0.08, 0.01) | 0.089 |
| WPAI – Work>0 | 0.19 (0.03) | (0.13, 0.24) | < .001 | 0.18 (0.17) | (0.15, 0.2) | < .001 | 0.01 | (-0.05, 0.07) | 0.75 |
| WPAI – Work | 0.07 (0.13) | (0.05, 0.08) | < .001 | 0.1 (0.03) | (0.06, 0.13) | < .001 | -0.03 | (-0.07, 0.01) | 0.175 |
| WPAI - Time Missed>0 | 0.1 (0.05) | (0, 0.2) | 0.042 | 0.2 (0.03) | (0.16, 0.24) | < .001 | -0.1 | (-0.2, 0.01) | 0.063 |
| WPAI - Time Missed | 0.01 (0.04) | (0, 0.01) | 0.059 | 0 (0.03) | (-0.01, 0.02) | 0.807 | 0.01 | (-0.01, 0.02) | 0.564 |

##

## **Supplementary table 8:** Model fit for both filtered and unfiltered models considering per-protocol analysis.

|  | **Fit** | | | | |
| --- | --- | --- | --- | --- | --- |
| **Outcome** | **Chi-sq (df)** | ***p*** | **RMSEA** | **CFI** | **SRMR** |
| Pain Level | 291.54 (2) | < .001 | 0.198 | 0.94 | 0.045 |
| GAD-7≥5 | 41.94 (2) | < .001 | 0.144 | 0.97 | 0.034 |
| GAD-7 | 9.56 (2) | 0.008 | 0.032 | 1 | 0.006 |
| PHQ-9≥5 | 19.78 (2) | < .001 | 0.112 | 0.98 | 0.026 |
| PHQ-9 | 5.3 (2) | 0.071 | 0.021 | 1 | 0.004 |
| WPAI – WPAI Overall>0 | 53.03 (2) | < .001 | 0.123 | 0.96 | 0.034 |
| WPAI Overall | 22.35 (2) | < .001 | 0.057 | 0.99 | 0.016 |
| WPAI – Activity>0 | 135.38 (2) | < .001 | 0.153 | 0.96 | 0.037 |
| WPAI – Activity | 74.16 (2) | < .001 | 0.099 | 0.98 | 0.023 |
| WPAI – Work>0 | 59.41 (2) | < .001 | 0.132 | 0.95 | 0.036 |
| WPAI – Work | 22.1 (2) | < .001 | 0.057 | 0.99 | 0.015 |
| WPAI - Time Missed>0 | 26.03 (2) | < .001 | 0.198 | 0.73 | 0.074 |
| WPAI - Time Missed | 7.99 (2) | 0.018 | 0.031 | 0.98 | 0.012 |
